# Potential Surprise Theory as a Practical and Theoretical Cornerstone of the Uncertainty‐Based Perspective on Risk

**DOI:** 10.1111/risa.70262

**Published:** 2026-05-14

**Authors:** James Derbyshire

**Affiliations:** ^1^ Chester Business School University of Chester, Queen's Park Campus Chester UK

**Keywords:** potential surprise theory, risk assessment, scenario planning, uncertainty

## Abstract

From all the knowledge that would emerge as relevant to it over infinite time, a risk analysis must be based on the cross‐section available at its undertaking. This creates a knowledge gap, which can lead to surprises. To address a similar problem in economic decision‐making, G. L. S. Shackle developed potential surprise theory (PST). PST's focus is on decisions that take the form of crucial, self‐destructive experiments, which destroy and radically remake the possibility space. Such decisions turn the kaleidic economy, the ceaseless shape‐shifting of which precludes absolute foreknowledge of all its possibilities. This dynamism means that the exhaustive listing of all possibilities required to assign probabilities necessitates the use of what Shackle called a “residual hypothesis” to represent all presently unknown scenarios. Yet, there is no way to know what probability to assign to this residual. PST overcomes this problem by employing a non‐probabilistic and nonadditive measure of uncertainty. PST has much to offer the uncertainty‐based perspective on risk, yet proponents of that perspective have been curiously inattentive to it. This article rectifies that by (1) showing how PST overcomes the residual‐hypothesis problem that is foundational to risk analysis; (2) juxtaposing PST and expected utility theory; (3) illustrating the nature of crucial experiments in risk analysis; (4) describing PST's language of possibility and its focus on surprises and extremes; and (5) discussing PST's operationalization in a risk analysis. In summary, PST can serve as a practical and theoretical cornerstone of the uncertainty‐based perspective on risk.

## 1 Introduction

Aven (2025) recently highlighted the ongoing transition towards an uncertainty‐based perspective in risk analysis. It has been underway for some time; many have contributed to it, and it has led to significant progress in terms of how uncertainty is handled in risk analysis. That said, given the uncertainty‐based perspective's focus on recognizing and preparing for potential surprises (e.g., Aven 2014; Paté‐Cornell 2012), it is curious that its proponents have paid so little attention to G. L. S. Shackle's extensive body of work on precisely that subject (Shackle 1949a, 1949b, 1949c, 1949d, 1950, 1951, 1952, 1953a, 1953b, 1955a, 1955b, 1958, 1959, 1961, 1967, 1970, 1972, 1979, 1980, 1983, 1984). This article addresses that lacuna by arguing that Shackle's potential surprise theory (PST) can serve as a practical and theoretical cornerstone of the uncertainty‐based perspective on risk.

The field's inattention to Shackle and PST is reflected in this journal. To be sure, Shackle and PST—or concepts originating in Shackle's PST, such as “unknowledge”^1^—were recently mentioned in this journal by Derbyshire (2022) and Derbyshire and Aven (2025). However, in neither case was PST the central focus. Employing this journal's website search engine to search for any occurrence of “G. L. S. Shackle” or “potential surprise theory” returns only those two papers. Moreover, using the same approach to search the *Journal of Risk Research, Reliability Engineering and System Safety*, and *Safety Science* returns no matches in each instance. Scholars of risk analysis have therefore all but entirely overlooked Shackle and PST. Indeed, there is even a paper in this journal that specifically focuses on the neglect of “potential surprises” within a probability‐based approach to risk analysis without ever referencing either Shackle or PST (Hammitt and Shlyakhtel 1999).

In PST, Shackle's concern was with how decision‐makers create history‐to‐come by making decisions representative of what he called crucial and self‐destructive experiments. Such decisions are crucial and self‐destructive because they destroy and radically remake the circumstances in which they are made (Shackle 1984). They are what turn the kaleidoscopic economy within a “kaleidic society” (Shackle 1972, 76), the ceaseless shape‐shifting of which precludes absolute foreknowledge of all its possibilities.^2^

Like the churn of shapes and patterns in a kaleidoscope, the economy has intervals of economic order, stability, and certainty. But they are interspersed by sudden cascades of disruptive change that destroy the present pattern and give rise to a new one, only for it too to be destroyed and replaced in turn, and so on unendingly (Shackle 1972). This kaleidic churn is set in motion by entrepreneurs’ decisions to launch a new business (Chiles et al. 2013) or by established businesses’ decisions to innovate a new product (Derbyshire and Giovannetti 2017), both of which unleash a cascade of responses and counterresponses (e.g., from competitors). This cascade destroys and radically remakes the possibility space that prompted the crucial decision by deleting some possibilities, generating new ones, and altering those remaining from before it was made.

In so doing, decisions constituting crucial and self‐destructive experiments pose a problem for traditional, probability‐based decision tools, which require the decision‐maker to close the future by listing all its possibilities. PST's usefulness for risk analysis stems, in part, from its ability to overcome the problems posed by probability's requirement to close the future. The importance of this matter for risk analysis is reflected in its having featured in the first full paper ever published in this journal. In that paper, Kaplan and Garrick (1981, 14, 15) imagine a critic commenting that,

A risk analysis is essentially a listing of scenarios. In reality, the list is infinite. Your analysis, and any analysis, is perforce finite, hence incomplete. Therefore no matter how thoroughly and carefully you have done your work, I am not going to trust your results. I'm not worried about the scenarios you have identified, but about those you haven't thought of. Thus I am never going to be satisfied.

Herein, we see writ large the problem of closing the future in risk analysis. A risk analysis focuses on known possibilities, but, as the critic correctly implies, uncertainty stems from the inevitability of previously unknown scenarios emerging over time that the analysis did not consider. Kaplan and Garrick (1981) acknowledge this criticism. The solution they suggest for enabling the assignment of probabilities by making a risk analysis exhaustive is a catch‐all residual category that represents all the scenarios left unconsidered (Kaplan and Garrick 1981).

Shackle would wholeheartedly agree with the critic. In setting out PST, he not only emphasized the problem of unknowledge—that is, what is *not* presently known—just as the critic does, but also proffered (what is in effect) the same solution to it that Kaplan and Garrick (1981) do: the use of a “residual hypothesis” (Shackle 1953a) to represent all the presently unknown possibilities^3^ left unconsidered by the analysis (Gilain et al. 2023). Yet, as Shackle (1955a, 1961) argued, and Kaplan and Garrick (1981) acknowledge, if such a solution is implemented within a probability‐based analysis, there remains the problem of what probability to assign to the residual. For that reason, Shackle considered the idea that the use of a residual can alone overcome the problem of unknowledge to be an “insidious fallacy” (Shackle 1961, 111; Derbyshire 2017, 81). For Shackle, the solution of a residual is only viable with a nonadditive^4^ measure of uncertainty.

In just one example of how the use of a residual is problematic within a probability‐based approach, because of uncertainty about the extent of unknowledge, and, therefore, about what probability to assign to the residual, an analyst may be susceptible to Catch‐All Underestimation Bias (Smithson and Ben‐Haim 2015; Tversky and Koehler 1994). The result would be to assign the residual an insufficiently large probability to cover all the possibilities that may emerge from it over time. If the analyst falls victim to this bias, over time, as new possibilities emerge, the only way to accommodate them is to change the probabilities assigned to those that *were* known and fully described by the analysis. This may result in highly consequential scenarios being assigned low probabilities, thereby affecting policymakers’ attentiveness to them (Derbyshire 2022).

There is no such problem when using PST because its measure of uncertainty (i.e., “potential surprise”) is non‐probabilistic and nonadditive. It does not require reserving an amount of uncertainty to accommodate presently unknown possibilities as they emerge over time. A residual can therefore be incorporated into the analysis without the risk of underestimating the likelihood needed to accommodate new scenarios as they emerge over time. This is a key benefit PST offers to risk analysis. It would be especially beneficial to risk analyses in what Cox (2020) calls “open‐world” contexts in which the problem of unknowns is particularly acute and novel scenarios can be expected to emerge over time.

The kaleidoscopic dynamism that necessitates a residual hypothesis remains problematic for risk analysis almost half a century after Kaplan and Garrick (1981). This problem can be neatly captured by borrowing from Shackle's unique lexicon: from all the knowledge that would emerge as relevant to it over the course of time, a risk analysis must inevitably reflect the cross‐section that is available in the “solitary present” (Shackle 1959, 286) moment of its conducting. This cross‐section may exclude important factors yet to be revealed or omit possibilities yet to be generated by the cascade of responses and counterresponses elicited by crucial experiments not yet conceived.

This problem is foundational to risk analysis, as evidenced by its discussion in the first full paper published in this journal (Kaplan and Garrick 1981). Despite the undoubted progress in accounting for uncertainty in risk analysis over recent decades, an adequate solution to it has yet to be provided. We hope to convince the reader that PST is at least part of the solution. The difficulty of providing a solution is reflected in the problem having two related but different sources. First, some knowledge that would influence a risk analysis is hidden or obscured during the analysis, only to be revealed over time. Second, subsequent developments (i.e., those that follow the risk analysis) generate a branching array of novel possibilities not included in the analysis. Moreover, the latter include subsequent developments set in motion by decisions made in response to the risk analysis itself, which trigger changes that negate the analysis that stimulated them. This introduces a recursivity that is a central component of uncertainty in its most extreme and difficult‐to‐grapple form.

A risk analysis sets in motion a cascade of responses and counterresponses, decisions, and interventions by multiple stakeholders, including regulators and those regulated.^5^ A risk analyst must therefore engage in what Knight (1921) called “twofold inference” (Townsend et al. 2024, 2025), which is best understood by reference to Keynes’ (1936) famous beauty‐contest example, in which participants are asked to choose the prettiest among six entrants. Crucially, only those who pick the one deemed the prettiest by the average of all respondents become eligible to win a prize (Derbyshire 2020). The analyst is therefore required *not* to consider the choice they would make in responding, but the choices others might make and how those choices might affect potential outcomes (Keynes 1936).

The consequences of the failure to consider twofold inferences can be quite stark. As Townsend et al. (2025, 16) succinctly state, “the failure to account for the recursive impact of choices to intervene, or not, in decision environments amplifies the problem of an unknowable future.” At the time of writing, the United States and Israel have chosen to intervene in Iran, with the apparent intention of mitigating the risk of Iran having nuclear weapons by changing its ruling regime. However, they may have failed to consider the possibility that Iran would respond by closing the Strait of Hormuz, which could have catastrophic consequences for the world economy.^6^ They may also have made the (currently, at least) seemingly wrong twofold inference that intervening to remove the leadership of the Iranian regime would lead the Iranian people to overthrow their government. This need to account for the recursive impact of intervention was also very evident during the COVID‐19 pandemic, in which it was necessary to draw twofold inferences about the behavioral responses to interventions designed to mitigate the pandemic's spread (e.g., social distancing), with little initial evidence on which to base these considerations (Derbyshire and Aven 2025).

Similarly, risk analysts and regulators at central banks are forever chasing their tails because financial institutions seek ways to circumvent financial regulations to maintain profitability. This makes the need for twofold inferences particularly stark in their domain. Analysts and regulators in central banks are engaged in a perpetual game of cat and mouse, requiring “fourth, fifth and higher degrees” (Keynes 1936, 156) of inference, due to the cascade of responses and counterresponses between regulators and those regulated; the kaleidic shape‐shifting of media and public attention; and the changes to sentiment they together produce, which drive unpredictable price movements (Shiller 2002). Or, to adopt another metaphor, central banks must play a perpetual game of whack‐a‐mole in which bashing down a risk by analyzing and regulating it causes the risk they successfully bashed down to change shape and reappear in a different guise elsewhere in the financial system, only for that version of it to be bashed down, in turn, by further analysis and regulation, only for it then to reappear once again in another shape and form elsewhere, and so on, ad infinitum.

Herein, we see both the kaleidoscopic shape‐shifting of risk and the recursivity that Knight (1921) saw as a central component of uncertainty in its most extreme and difficult‐to‐grapple form. Coping with this requires a highly flexible, nonadditive measure of uncertainty, which is just what PST possesses. We therefore invite the reader to peer through Shackle's kaleidoscope for a wholly different perspective on risk and how to handle it. Using coronavirus decision‐making as an example, Section 2 illustrates how the traditional perspective on risk downplays uncertainty. Section 3 describes the specific problem of surprises from unknowns. Section 4 describes PST's language of possibility and its non‐probabilistic and nonadditive measure of uncertainty. Section 5 argues for PST as a cornerstone of the uncertainty‐based perspective on risk and how it might be operationalized in a risk analysis. Section 6 provides some concluding remarks.

## 2 The Traditional Perspective on Risk

### 2.1 The Traditional Perspective on Risk and Its Downplaying of Uncertainty

Aven (2025) refers to the traditional perspective on risk as “*E*[*X*]‐based thinking” to reflect its focus on calculating expected values. To understand how *E*[*X*]‐based thinking constrains and downplays uncertainty, assume that *X* represents a quantity of interest, such as the number of fatalities over 10 years from a pandemic. *E*[*X*]‐based thinking focuses attention on the expected value of *X* (i.e., *E*[*X*]) or on a combination of *X* and its associated probabilities *P*, denoted as (*X*, *P*) and represented by a probability distribution (Aven 2025). Under this traditional perspective on risk, decisions to mitigate risk are optimized by fully listing all possibilities and calculating their expected values.

However, for several reasons, this perspective on risk downplays the full extent of uncertainty. For most risks, the primary concern is not the theoretical value *E*[*X*] or the uncertainties associated with its estimation. Instead, the primary concern is the specific value of *X*, for example, the actual number of pandemic fatalities and the associated uncertainties (Aven 2025). In contrast, *E*[*X*] represents an average across an infinite number of activities or events similar to that considered (e.g., infinite pandemics). Focusing on *E*[*X*], therefore, downplays uncertainty because the uncertainty associated with *E*[*X*] is typically much lower than that associated with *X* itself.

Moreover, at least in its standard form—and notwithstanding the use of a residual to encompass all other presently unknown scenarios (Kaplan and Garrick 1981), which does not alone solve the problem of unknowledge for reasons elaborated on shortly—*E*[*X*]‐based thinking also downplays uncertainty by requiring an exhaustive listing of all possibilities. This requirement implies, for example, that the outcomes from decisions to intervene to mitigate risk are fully knowable in advance. This, in turn, implies that such decisions do not produce unpredictable changes to behavior or irreversible outcomes that radically remake the possibility space. In short, the assumption in *E*[*X*]‐based thinking is that the possibility space informing the making of a decision is updated over time rather than radically remade by the decision itself. As demonstrated by the case of COVID‐19 decision‐making in the United Kingdom (UK), that is a dubious assumption.

### 2.2 The Traditional Perspective on Risk and Its Downplaying of Uncertainty: An Illustration

When COVID‐19 reached the UK in early 2020, the government did not implement an immediate and strict quarantine (Adam 2020; Cairney 2021). Instead, it attempted to model the “actuarial considerations,” as Shackle (1955a, 3) calls them (Derbyshire 2022). Several authors have criticized this decision to model the pandemic's spread first, noting that the models used for this purpose underestimated the *R* rate of transmission and, therefore, the doubling time of cases, and have blamed it for the 2‐week delay in implementing a strict quarantine (Cairney 2021). It has been estimated that, had the decision to implement a strict quarantine been made just 1 week earlier, it would have saved 23,000 lives (Dyer 2025). Therefore, because a virus's spread would tend to be exponential, delaying that decision by 2 weeks may have cost many more than 50,000 lives.

This 2‐week delay in implementing a strict quarantine exemplifies the problematic nature of *E*[*X*]‐based thinking, or what might alternatively be termed the *E*[*X*]‐based mindset. In *E*[*X*]‐based thinking, the focus is on presently available knowledge rather than change over time, and attention is directed away from the potential for surprises and extreme impacts. There was no way to know whether the *R* rate of transmission was accurate at the very start of the pandemic and no reason to think it would remain at that level even if it was. Therefore, modeling the rate of transmission using the then–current estimates, which had dubious evidentiary foundations, could have given a misleading impression of the doubling time of cases (which would likely decrease over time anyway) and related metrics, such as intensive care admissions. Which is exactly what it did (Derbyshire and Aven 2025).

The decision not to implement an immediate and strict quarantine is not only an example of *E*[*X*]‐based thinking's focus on what is perceived to be known at the expense of what is unknown (Derbyshire and Aven 2025). It is also an example of a decision of the type Shackle called a “crucial and self‐destructive experiment” (Shackle 1949b, 6). Such decisions are “non‐seriable” and “non‐divisible”^7^ because they are unique, unrepeatable, and irreversible (Shackle 1952, 23–24). They do not update but destroy and remake the space of possibilities by deleting some possibilities from it, generating and adding new possibilities to it, and altering those that remain in it from before the decision was taken. The UK government's erroneous decision to delay implementing a strict quarantine was a decision representative of a crucial experiment that irreversibly transformed the circumstances in which it was made (Derbyshire 2022).

Of course, a later decision to implement a strict quarantine could still have been made and was. But it was not made under the same circumstances. Because a virus's spread is exponential, once sufficiently unleashed, it becomes irreversible. The decision not to immediately implement a strict quarantine, therefore, destroyed and remade the space of possibilities. Some of its possibilities were indeed updated, while others were deleted, and new ones were added. For example, it deleted the possibility of containing the outbreak with minimal harm (Derbyshire 2022).

Decisions in the form of crucial experiments turn the kaleidoscope, destroying the pattern into which its shapes had previously fallen and replacing it with another, very different pattern. Once gone, the pattern never reemerges in exactly the same form, so no similar future decision can ever be made under those same circumstances. It was just such decisions, which feature a high degree of unknowledge and take the form of crucial and self‐destructive experiments, that were Shackle's concern in setting out PST. In the next section, we elaborate on why using probability is problematic when it comes to decisions of this type.

## 3 The Fundamental Problem of Surprises From Unknowns

### 3.1 Subjective Probability and the Problem of Unknowns

In response to what has preceded, it may be argued that the nature of a decision as a crucial self‐destructive experiment may well preclude the use of frequency‐based probability in decision‐making. Of course, a sample of cases on which to calculate a frequency‐based probability cannot be constructed for a unique decision that lacks any element of seriability and divisibility, such as that about a strict quarantine at the start of COVID‐19. But this problem does not apply to subjective forms of probability, such as Bayesian learning and inference, which is often used to estimate the probabilities needed to calculate the expected values that are the output of the *E*[*X*]‐based approach. In our previous description of the COVID‐19 decision‐making example, we already provided logic showing that this argument does not hold. We have suggested that subjective probability, when expressed through Bayesian learning and inference, also has difficulty dealing with kaleidic dynamism and the unknowledge it generates. In this section, we expand on the limitations of Bayesian learning and inference in the face of unknowns, using the example of Feduzi and Runde (2014).

Consider someone attempting to estimate the proportion of red balls in an urn she believes contains only red and black balls, who, after drawing only red and black balls for a time, then draws a yellow ball. If Bayesian learning and inference had been used to estimate the probabilities underlying the expected‐value calculation in this example, the estimation process would now grind to a halt. That is because the Bayesian approach precludes adding new states or updating a prior probability of zero to a positive posterior probability.

On the basis of the logic of Bayesian learning and inference, conditionalizing on information that a previously unarticulated possibility has been introduced is nonsensical^8^ (Feduzi and Runde 2014). Such conditionalization presupposes a well‐defined prior probability for that possibility (Earman 1992; Feduzi and Runde 2014). Upon encountering the surprise of drawing a yellow ball, a decision‐maker using Bayesian learning and inference would be obliged to start over with a reformulated set of possibilities, respecify the priors, and resume sampling and updating. Moreover, this would have to be repeated whenever the decision‐maker encounters a state (i.e., a differently colored ball) previously unknown to her (Feduzi and Runde 2014). Herein, we see the difficulty that the Bayesian approach faces in handling the emergence of previously unknown possibilities over time. However, as we elaborate on next, this problem of unknowledge does *not* apply only to subjective probability in the form of Bayesian learning and inference but to any form of subjective probability, as all place emphasis on knowledge rather than unknowledge.

### 3.2 Expanding the Bounds of Possibility Into the Unknown Through Generative Rationality

Following the above example, a critic may respond that although Shackle's criticisms of probability may therefore apply to the Bayesian version of subjective probability and not only to frequency‐based probability, a simple subjective probability based on available knowledge can be assigned to any event, including a unique one (Singpurwalla and Wilson 2008). This would merely require that the analyst assign a probability based on her judgment, as informed by the knowledge possessed at the time of the assignment. If the event to be assigned a probability is unique, it need not be grouped with alternative possibilities that must be additive and sum to unity. The key point is that a unique event can be assigned a subjective probability without the need to accommodate new possibilities over time. Any criticisms related to probability's additivity and the difficulty of accommodating emergent possibilities over time are therefore irrelevant.

That may be so, but the probability still focuses on presently available knowledge. Whereas the primary concern in PST is uncertainty from unknowledge and, therefore, the generation of alternative possibilities through acts of imagination.^9^ These may be informed by but are certainly not exclusively dependent on presently available knowledge. This generative aspect of PST, grounded in acts of imagination, is critical to understanding the value of Shackle's approach and its sharp contrast with *E*[*X*]‐based thinking.

Indeed, the “generative rationality” (Gilain et al. 2023) underpinning Shackle's approach is currently receiving much attention in the fields of management, strategy, and innovation, especially in research on theory‐driven strategizing (Camuffo et al. 2024; Gilain et al. 2023; Felin and Zenger 2017; Hatchuel 2023; Le Masson et al. 2019). In that research, the emphasis PST places on generating novel scenarios that expand a possibility space into the presently unknown through acts of imagination has been explicitly juxtaposed against the traditional view of rationality in *E*[*X*]‐based thinking, which instead emphasizes the optimization of already known possibilities (Ehrig and Foss 2022; Hatchuel 2023). Gilain et al. (2023) illustrate the difference between these two understandings of rationality as follows.

Imagine a self‐driving car controlled by an algorithm. Suddenly, the brakes fail as the car approaches a busy pedestrian crossing, and the algorithm must decide which option to take. The options are to kill the pedestrians on the crossing or to save them by crashing the car into a wall, killing its occupants instead. This presents a dilemma,^10^ especially for algorithms that make decisions based on the traditional view of rationality as optimizing known possibilities. It seems odd to talk of optimizing when neither of the known options is particularly desirable. If time permitted, the strong temptation (at least for a human decision‐maker) would be to generate an alternative, novel, and more desirable, but presently unknown, option that would lead to a superior outcome.

In describing this example, Le Masson et al. (2019) explicitly associate the dilemma it presents with expected utility theory (EUT), or what we have termed *E*[*X*]‐based thinking. EUT fails to recognize that rationality is not solely about optimizing possibilities based on currently available knowledge. It is also about generating novel possibilities by expanding knowledge into the domain of the presently unknown. Gilain et al. (2023), therefore, ask what a decision‐maker should do in circumstances in which she suspects the set of possibilities she is considering is incomplete and could be augmented by other, presently unknown possibilities that are difficult to imagine.

Essentially, this is a problem of uncertainty about the bounds of a possibility space. What should be deemed within the realm of the possible and what should be excluded from it? In a complex and kaleidic world featuring “open‐world novelty” (Cox 2020), robustly establishing the bounds of a possibility space is crucial to reducing a risk analysis's susceptibility to surprises. Set the bounds of possibility too narrowly, and one increases susceptibility to surprises in the form of possibilities that lie beyond those bounds. However, equally problematic, setting the bounds too broadly undermines the credibility of the risk analysis, which may make policymakers inattentive to its implications.

The key point is that Shackle's PST is based on a generative rationality that emphasizes the expansion of a possibility space into the presently unknown through acts of imagination. Subjective probability emphasizes presently available knowledge. In a complex, kaleidic world characterized by emergence and novelty, this emphasis on what is presently known will increase susceptibility to surprises. Generative acts of imagination are needed to overcome this problem and are central to PST.

### 3.3 Capturing Presently Unknown Possibilities in a Residual

As the growing body of research on generative rationality discusses (Gilain et al. 2023), Shackle addressed the problem of the emergence over time of presently unknown possibilities by proposing a “residual hypothesis” (Shackle 1953a) designed to cover all the possibilities left unconsidered by an analysis. This solution is similar to that suggested by Kaplan and Garrick (1981) in response to their imaginary critic's complaint that a risk analysis could never provide an exhaustive set of scenarios.

However, there is a fundamental point of departure between these two similar solutions. Kaplan and Garrick (1981) suggest that a catch‐all residual category for “other” scenarios can address the problem of unknowns within a probabilistic approach. Shackle is explicit in suggesting that it cannot work if probability is retained as the measure of uncertainty (Katzner 1986). In Shackle's view, using a residual only solves the problem of surprises from unknowns if a nonadditive measure of uncertainty is used, which he provides in the form of potential surprise. Understanding why Shackle (1972) thought the solution of a residual required a nonadditive measure of uncertainty is aided by reference to Gilain et al.’s (2023) simple formalization.

Gilain et al. (2023, 666) describe Shackle (1972, 22) as stating that the residual gathers the events that are “unthought of and incapable of being envisaged before the deadline of the decision has come.” We can relate this to the deadline for a risk analysis. As Kaplan and Garrick's (1981) imaginary critic correctly implies, the risk analysis will inevitably exclude some presently unknown possibilities because they are not represented in the analyst's knowledge set at the point of that deadline.

Following Gilain et al. (2023), Shackle's residual hypothesis can be formalized as ε, representing what are then (i.e., at the time of the risk analysis) presently unknown events or outcomes. The universe of all possible events and outcomes, represented by Ω, then corresponds to the union of known events and outcomes $\theta_{i}$ and the set of unknown events and outcomes represented by the residual, as in $\cup_{i=1}^{m}\theta_{i}\cup \varepsilon$ = Ω. However, of course, the analyst could simply assume ε to be negligible. Indeed, exactly that assumption is the basis for Savage's (1951, 1954) “small‐world representation” (Derbyshire and Aven 2026; Feduzi et al. 2022), which is foundational to EUT, and therefore to *E*[*X*]‐based thinking. Under this assumption of there being negligible unknown scenarios, EUT and Bayesian learning and inference can together be used to explore a possibility space reduced to ${\left\{\theta_{i}\right\}}_{i=1\text{…}m}$ (Gilain et al. 2023, 666).

However, in contrast to Savage (1951, 1954), the logic underpinning Shackle's rejection of probability is that, in the majority of decision contexts, the set of unknown events and outcomes ε is far from negligible. Indeed, Shackle considered unknown events and outcomes to be predominant in many, if not all, decision contexts (Derbyshire 2017). That may or may not be an exaggeration, but they are certainly predominant in some open‐world contexts (Cox 2020). Shackle's view is that, over the course of time, some of these unknown possibilities will emerge and significantly disturb the small‐world representation that is ${\left\{\theta_{i}\right\}}_{i=1\text{…}m}$(Gilain et al. 2023). Under such circumstances, the logic of EUT breaks down, as illustrated by Feduzi and Runde's (2014) example of unexpectedly drawing a yellow ball from an urn that was thought to contain only black and red balls.

For two further and final reasons, Kaplan and Garrick's (1981) solution of a residual category representing all “other” scenarios does not help if the measure of uncertainty is a probability. First, because, as Shackle (1955a, 1961) discusses and Kaplan and Garrick (1981) acknowledge, it still leaves open the question as to how much probability to assign to that residual. Second, and relatedly, as events unfold over time to reveal the unknown scenarios previously hidden within it, if the amount of probability assigned to the residual is too little to accommodate them into the analysis, the only way to accommodate the revealed scenarios is to change the probability assigned to those lying outside the residual that *were* known and fully described. This change may have the unfortunate consequence of decreasing the probability assigned to highly consequential outcomes, which may mean they do not receive the attention their potential impact warrants (Derbyshire 2022).

Moreover, there is much potential to underestimate the probability assigned to the residual due to “Catch‐All Underestimation Bias” (Smithson and Ben‐Haim 2015; Tversky and Koehler 1994), which can occur when events are combined into a single superset (e.g., a residual hypothesis). Under this bias, the probability assigned to the superset (i.e., the probability of any member of it occurring) is less than the sum of the probabilities assigned to its constituent categories of event (Derbyshire 2017). An example is someone asked to estimate the probability of being delayed tomorrow, who then assigns a lower overall probability to this than the sum of the probabilities they would individually assign to being late out of bed, delayed by traffic, distracted by meeting someone on the way, and so on (Smithson and Ben‐Haim 2015). Having set out these problems with probability as a device for dealing with uncertainty, we now turn to describe the solution: PST.

## 4 A Synopsis on PST

### 4.1 Measuring Belief as Disbelief

In setting out PST, Shackle viewed belief in a possibility as simply corresponding to “the degree of surprise to which this belief exposes us and will subject us in case the hypothesis proves false” (Shackle 1949a, 9; Zappia 2014, 1138). Therefore, because it emphasizes the degree of surprise we experience when a hypothesis is proved *false*, PST has a decision‐maker consider their degree of *disbelief* in alternative possibilities, rather than their degree of *belief*, as subjective probability would. This inverted approach may seem absurd to someone imbued with *E*[*X*]‐based thinking, which emphasizes knowledge. But it makes perfect sense if the focus is on unknowledge and the need is to accommodate novel possibilities that emerge over time without affecting those already known and considered. Because one can disbelieve in as many alternative possibilities as one may like, the degree of uncertainty assigned on the basis of that disbelief does not need to be additive like probability does. The degree of disbelief in one possibility has no bearing on the degree of disbelief in another.

Shackle therefore saw the problem with belief based on knowledge as its tendency towards exclusion. Although one can only really believe wholeheartedly in a single possible outcome, one can disbelieve wholeheartedly (or to different degrees) in the possibility of a whole raft of alternatives. Measuring belief with probability implies that there must be an outcome, or a set of outcomes, that will occur with certainty. No other possibility outside of that set could occur. Under subjective probability, if one truly believes in a possibility, one must assign it unity (i.e., 100%), thereby excluding any other possibility. Shackle considered such certainty misleading in most decision contexts and did not understand why belief in one outcome should necessarily affect that in another, as it must under probability's additivity axiom (Basili and Zappia 2009a, 2009b).

To arrive at a measure of “disbelief,” Shackle envisages individuals assigning degrees of surprise ranging from zero for outcomes that seem perfectly possible because there is little or nothing standing in their way to a maximum value representing complete astonishment—say, for example, 10—for outcomes that the decision‐maker wholeheartedly disbelieves because, under current conditions (i.e., given the present state of knowledge), they seem impossible (Basili and Zappia 2009a). Appendix 1 contains a complete axiomatization of this measurement process drawn from Shackle (1961, 79–85).

### 4.2 Interpreting the Axioms of PST

Interpreting PST's axioms: to assign different degrees of potential surprise to alternative possibilities, Shackle envisages individuals as imagining what the “sequels” to their decisions and actions may be by asking themselves, “How surprised would I be if this outcome actually occurred, if, at the time it occurred, I were still looking at the world in the way I look at it right now?” (Earl and Littleboy 2014, 88). One answer could be “Not at all surprised, this seems perfectly possible,” or it could instead be “Very surprised indeed, there is just so much standing in its way” (Earl and Littleboy 2014, 88). Through this means, PST prompts decision‐makers to consider alternative outcomes through a natural language of possibility.

Rather than focusing on its drivers, because of its emphasis on surprises, which are dashed expectations, PST focuses on the obstacles standing in the way of an outcome's realization. Here we again see how PST focuses on uncertainty by inverting perspective, just as it does by focusing on disbelief rather than belief. The focus shifts from the realization of the expected to the realization of the unexpected, in the form of an outcome the decision‐maker does not expect, given their view of the world as it is presently. We see here how the ontology of PST is very different from that in *E*[*X*]‐based thinking.

In selecting among alternatives, the decision‐maker bases the choice on the attention‐arresting power of pairs of “focus outcomes,” which are the most extreme plausible outcomes on the positive and negative sides (Derbyshire 2017). This attention‐arresting power depends both on an outcome's value or payoff and on the potential surprise attached to it (Foldes 1958). The two focus outcomes are therefore those that, in their combination of possibility (i.e., potential surprise) and value (i.e., payoff), are strikingly favorable and unfavorable in the decision‐maker's mind. The attention‐arresting strikingness of these two focus outcomes exceeds that of all the alternatives for that decision‐maker; what is attention‐arresting to one person may not be to another.

As Foldes (1958) notes, potential surprise is an entirely psychological and subjective measure. The scale on which potential surprise is measured is not linked to an underlying objective measure, such as drawing balls from an urn. This, again, is in keeping with the focus on unknowledge and uncertainty. As noted earlier, PST is based on “generative rationality” (Gilain et al. 2023), which is fundamentally different from rationality understood as the optimization of known possibilities, as in EUT. The emphasis in PST is on expanding the possibility space into the presently unknown by generating new possibilities through imagination‐based experimentation (e.g., envisaging alternative scenarios). This requires an open, flexible measure of uncertainty that is properly subjective, unlike a subjective probability that is tethered to an objective measure (e.g., balls drawn from an urn or odds‐based betting).

In PST, the decision‐maker is viewed as making “bets” by comparing pairs of alternative focus outcomes, rather than on the basis of odds. Adapting from Derbyshire (2017, 80), the process of making a decision in PST can therefore be summarized as follows:

1. For the particular decision under consideration—for example, whether to start a business of one type or another, to innovate a new product, to build a submarine‐based or land‐based nuclear deterrent, to decide which existential risks to invest in mitigating, and so on—imagine a set of alternative possibilities and their outcomes.
2. For each outcome, consider whether its actualization is perfectly possible, somewhat surprising, highly surprising, impossible, and so on, assuming that the world remains as one sees it presently. Assign a measure on a “surprise scale” (e.g., 0–10) accordingly for that outcome. Zero is for outcomes deemed perfectly possible, and 10 is for outcomes deemed impossible, with degrees in between representing varying degrees of surprise, where a degree closer to 10 indicates greater disbelief in an outcome's possibility and a degree closer to 0 indicates greater belief in its possibility.
3. For each outcome, imagine the impact (i.e., the gains or the losses) should it be actualized.
4. For each alternative decision, identify the single outcome that is most arresting because of the combination of its potential surprise and its positive payoff, and the single outcome that is most arresting because of the combination of its potential surprise and its negative payoff. These are the most extreme yet still plausible “focal outcomes” on the positive and negative sides, respectively. For each alternative decision, this therefore results in a pair of focus outcomes, formally defined as the maximization of a continuous stimulation function, representing potential gains and losses, subject to a continuous potential surprise function, representing uncertainty (Zappia 2014, 1139).
5. For each alternative decision, compare the respective pairs of focus outcomes in light of the respective (and subjective to the individual decision‐maker) attitude towards the trade‐off between losses and gains (Earl and Littleboy 2014, 98). Select the decision for which this trade‐off is the most compelling.

In the remainder of this section, we set out an illustration related to business investment, then discuss PST in relation to the COVID‐19 example that framed the earlier discussion of the deficiencies of *E*[*X*]‐based thinking.

### 4.3 Making Decisions Using PST: An Illustration

**FIGURE 1:**
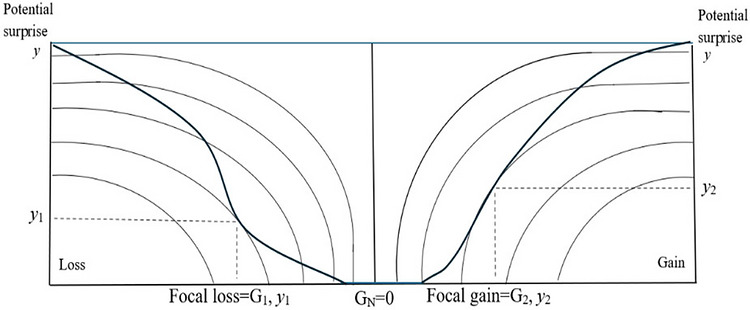
Potential surprise theory's ascendancy function and a choice curve. Adapted from Shackle (1961, 149, 155).

On the basis of the preceding discussion, the measure of disbelief that is potential surprise is combined with a decision‐maker's anticipated gain or loss from a choice to inform decision‐making. By combining these two elements—potential gains and losses and the degree of disbelief in them as measured by potential surprise—the decision‐maker ranks choices based on the desirability of each alternative's pair of focal outcomes. Figure 1 illustrates what Shackle called an ascendancy function. It highlights in bold a single curve representing one choice or action and shows its two focus outcomes, which we have stated to be those resulting from maximizing a continuous stimulation function subject to a continuous potential surprise function (Zappia 2014).

At one point in Figure 1, the boldly highlighted choice curve lies flat against the horizontal axis because the outcomes at that point are each considered “perfectly possible.” They therefore have a potential surprise of zero. Imagine, for example, that this curve represents one possible choice for an entrepreneur deciding which business opportunity to invest in. That opportunity requires an investment of $5000. Imagine that the scale on the vertical axis of Figure 1, representing potential surprise, ranges from 0 at its juncture with the horizontal axis to 10 at *y*. The arresting loss for the considered investment is therefore $10,000 (*G*_1_) and is assigned a degree of potential surprise of approximately 3 (*y*_1_). In combination, *G*_1_ and *y*_1_ represent the most arresting loss, which the entrepreneur believes cannot be exceeded for this investment, regardless of the scenario that follows. The arresting gain, however, is $25,000 (*G*_2_) and, as a rough approximation, has been assigned a degree of potential surprise of 4 (*y*_2_). In combination, *G*_2_ and *y*_2_ therefore represent the most arresting gain, which the entrepreneur believes cannot be exceeded for this investment, regardless of the scenario that follows (i.e., regardless of the state of the world that later emerges).

The decision‐maker would consider several such choice curves associated with different investment options; Figure 1 illustrates just one. A choice between alternatives is made by comparing, for each alternative, pairs of focal outcomes, just like the pair illustrated. These are made comparable through standardization across alternatives (Cantillo 2015). In this way, it becomes possible to compare and rank decisions based on how arresting their potential gains and losses are, as modified by the degree of disbelief (i.e., potential surprise) in their occurrence. Different values of the scalar variable of interest, which is in this case monetary, are associated with different degrees of disbelief (Cantillo 2015), and together they establish the feeling of surprise by anticipation: the potential surprise (Cantillo 2015).

This places the focus of decision‐making squarely on the best‐ and worst‐case scenarios, which is desirable when a decision would have irreversible impacts, as in the case of implementing a strict quarantine at the start of the COVID‐19 pandemic. At the start of the COVID‐19 pandemic in the UK, because of this focus on arresting outcomes and best‐ and worst‐case scenarios, the use of PST may have prompted the invocation of the precautionary principle (Aven 2011), resulting in the immediate implementation of a strict quarantine. This would have saved many lives, as we outlined earlier. Below, we elaborate further on how PST might have been used in the context of COVID‐19 decision‐making.

### 4.4 Making Decisions in a Multi‐Criteria Context

An observant reader might respond to the above explanation by commenting that it is all well and good when there is a single and common unit of measurement, such that the decision‐maker simply has to compare gains and losses in terms of that single and common unit of measurement. In the outlined example in Figure 1, the most arresting outcomes both pertain to just one variable, which is monetary for both the focal loss and the focal gain, making them directly comparable. Moreover, in the example associated with Figure 1, the alternative choice curves would also share the same unit of measurement (i.e., the payoff from an investment in dollars) and could therefore be directly compared with the choice curve highlighted in Figure 1. The simplicity of the example serves to communicate the PST approach, but does it do so at the expense of being unrepresentative of most decision‐making contexts? Moreover, is it especially unrepresentative of decisions in “open‐world” contexts (Cox 2020) in which there may be many decision criteria at play simultaneously?

Consider once more, for example, the context of COVID‐19 with which we framed our earlier discussion on the deficiencies of *E*[*X*]‐based thinking. Of course, the common unit of measurement in that case could be fatalities, or perhaps admissions to intensive care, or some other metric that could uniquely represent both losses and gains from alternative choices, actions, and decisions. However, whether or not it jars with one's ethics, the effect of a strict quarantine on economic output will inevitably have been taken into consideration in COVID‐19 decision‐making. In the UK's case, that consideration may even have been what motivated the delay in implementing a strict quarantine, whereas modeling was undertaken (Adam 2020). This would imply that fatalities, or perhaps intensive care admissions, were one decision‐making criterion, and the effect on economic output was another. What, then, if there are more criteria to be considered than in the simple example associated with Figure 1?

For example, a decision‐maker might have considered a “do nothing” scenario in which the virus spreads throughout the whole population of the UK. Moreover, as part of that scenario, she may even have anticipated that the proportion of cases requiring admission to intensive care could be higher than the evidence available early in the pandemic's unfolding implied, as indeed was the case (Adam 2020). Assuming that health services could accommodate that number of admissions, consideration may then have turned to the economic cost in an alternative “strict quarantine” scenario. When comparing these scenarios and their choice curves, there might therefore have been at least two variables at play, with uncommon units of measurement: the number of patients admitted to intensive care and the loss of economic output, with the latter perhaps measured as the percentage by which GDP shrank (e.g., −10% or −20%). There may have been still other criteria of importance to the decision, as associated with still other scenarios, as represented by other choice curves. How can these be made comparable when their units of measurement differ?

Shackle never directly addressed such circumstances, but others have discussed them in relation to PST (Earl 1986). We will not go into detail here, as the purpose of this article is simply to persuade the reader that PST should be considered an important tool within the uncertainty‐based perspective on risk. Suffice to say here, briefly, that one might envisage a decision‐maker as having a hierarchy of objectives or criteria and progressively considering options in relation to each of them according to their priority through a nested and iterative analysis, thereby arriving at a choice for which the most arresting focal outcomes were deemed acceptable across all levels and criteria of analysis.

## 5 PST as a Theoretical and Practical Cornerstone of the Uncertainty‐Based Perspective on Risk

### 5.1 Overcoming the Difficulties Associated With Using a Residual

In summarizing why PST can serve as a practical and theoretical cornerstone of the uncertainty‐based perspective on risk, reconsider the problem of using a residual, which we earlier described as foundational to risk analysis, as reflected in its discussion in the first issue of this journal. It is hopefully clear to the reader from what has preceded that, in PST, new possibilities can be considered as the decision‐maker becomes aware of them or as subsequent developments generate them, without affecting the uncertainty (i.e., potential surprise) assigned to those already known and considered.

Of course, newly revealed information or subsequent developments might also affect those known possibilities, requiring their revision or altering the ranking of alternative choices based on them. But that would be because the new information or developments affect the underlying reasoning on which they are based, in turn, perhaps also affecting their payoff and degree of potential surprise. If changes to reality that generate new possibilities or new information do not affect the underlying reasoning of already known and considered possibilities in any way, then there is no need to revise the uncertainty assigned to them to accommodate new possibilities. That is because the measure of uncertainty, potential surprise, is nonadditive.

As previously unknown possibilities emerge from a residual over time, they can therefore be easily accommodated in the analysis. Moreover, there is no problem accommodating changes in the degree of potential surprise assigned to the residual, which can vary as the world changes over time, revealing its secrets and generating new possibilities, without affecting the degree of uncertainty assigned to the known possibilities lying outside the residual.

### 5.2 Journeying From the Traditional to an Uncertainty‐Based Perspective on Risk

Our earlier discussion distinguished between the traditional perspective on risk, characterized as *E*[*X*]‐based thinking, and an emerging uncertainty‐based perspective. The latter focuses directly on *X* and its potential values, recognizing that these values may change over time and may include extremes that extend well beyond past outturns or what is conceivable given presently available knowledge. This leads to a different understanding of risk that not only includes but also explicitly emphasizes novel and extreme possibilities and highly consequential, irreversible impacts.

By making *X* and its uncertainties the direct focus of analysis, the proponents of the uncertainty‐based perspective on risk seek to make the possibility of surprises more explicit (Aven 2014, 2025; Derbyshire et al. 2025; Paté‐Cornell 2012). As our discussion has shown, PST is closely aligned with the ethos and purpose of the uncertainty‐based perspective on risk because it places the focus squarely on *X* and its uncertainties and is explicitly concerned with surprises, extremes, and unknowns. Indeed, it places the focus of decision‐making squarely on surprises in the form of extreme, arresting outcomes, and it emphasizes unknowledge over knowledge.

Shackle laid PST's foundations at approximately the same time that von Neumann and Morgenstern (1944) and Savage (1951, 1954) were laying the foundations of EUT, which underpins *E*[*X*]‐based thinking and more traditional perspectives on risk. In the immediate decades that followed, as EUT was gaining ascendancy, Shackle engaged little with this rival approach (Earl and Littleboy 2014). This may have been because Shackle considered himself embarked on a wholly different journey, whose destination was a method for dealing with uncertainty of a type very different from that in EUT. That wholly different journey is evident in PST's “generative rationality” (Gilain et al. 2023; Le Masson et al. 2019), which emphasizes expanding the possibility space into the presently unknown through acts of imagination and which is very different from rationality understood as the optimization of already known possibilities, as in EUT. In short, Shackle had a very different ontology from that of von Neumann and Morgenstern (1944) and Savage (1951, 1954), and this is evident in the differences between PST and EUT.

This fundamental ontological difference is also evident in the assumption that unknowns are negligible in the foundational texts of *E*[*X*]‐based thinking (Savage 1951, 1954). By contrast, in many risk‐analysis contexts, they are far from negligible. Society is a complex, adaptive, and open system composed of a myriad of nested complex, adaptive, and open systems at all levels (Buckley 1968). The futures of such systems are rarely, if ever, closable, meaning their future possibilities can never be exhaustively listed. This is not least because such systems involve human actors who act reflexively to adapt their decision‐making and strategizing in light of new information that is revealed over time (Derbyshire 2016), or on the basis of others’ actions and expectations, or simply on the basis of the “uncaused cause” of human motivations and whimsy (Shackle 1983). In many contexts of interest to risk analysts, the primary factor giving rise to the necessity of a residual and, therefore, also to the need for a nonadditive measure of uncertainty is the unpredictability of human behavior, motivation, and whimsy. This contrasts with the stability implied by EUT's requirement to list all possibilities in advance.

During the early postwar decades, and despite Shackle's lack of engagement with EUT, PST was EUT's only meaningful rival (Basili and Zappia 2009a). In more recent decades, their paths have diverged, and PST has slipped into obscurity as EUT has come to dominate. Yet each new surprise we are subjected to further calls into question that dominance, returning PST to prominence. Shackle was a pathfinder on the journey towards a better accounting of uncertainty; he was decades ahead of his time. Scholars of risk analysis, in their journey from the traditional to an uncertainty‐based perspective on risk, are treading in the footsteps left by Shackle. His journey was a lonely one through terrain that became increasingly hostile to any conception of uncertainty that recognized it as anything other than probabilistic. We owe it to G. L. S. Shackle to accompany him belatedly on his journey by giving PST the attention it deserves within the uncertainty‐based perspective on risk.

### 5.3 Operationalizing PST in Risk Analysis

What has preceded in this article should not be taken to imply that PST can replace risk analysis or that it should necessarily replace any specific tool used to carry it out, whether associated with the traditional or the uncertainty‐based perspective on risk. The primary purpose of this article has been to persuade the reader that, given the journey towards greater recognition of and accounting for uncertainty in risk analysis, as has been underway for some time, overlooking PST is an important oversight that should be rectified. It is hoped that this article might stimulate others to look at risk through Shackle's kaleidoscope. PST can provide the field with a practical decision‐making tool and an underlying philosophy that recognizes the uniqueness and irreversibility of many developments over time.

There is a practical tool that loosely approximates PST that can operationalize it in risk analysis. For many decades, Royal Dutch Shell has used scenario planning to consider the best‐ and worst‐case boundaries of the possible trajectories of its external environment (Earl 2023; Jefferson 1983, 2012, 2014). In his private correspondence, Shackle stated that he considered the version of the intuitive logics (IL) scenario‐planning method used at Royal Dutch Shell in the 1970s and 1980s to be PST's practical manifestation (Derbyshire 2017; Derbyshire, Feduzi, et al. 2023). Modern versions of IL, use of which has spread widely beyond the bounds of Shell, have departed somewhat from how it was originally used at Royal Dutch Shell at that time (Derbyshire 2023), but the family resemblance remains evident in that the scenarios they generate are framed by two sets of focal outcomes, which, respectively, bookend the axes of the 2 × 2 matrix from which scenarios are derived. Indeed, this modern version of IL has been enhanced by a recent modification, as set out and tested in a randomized controlled trial in this journal (Derbyshire et al. 2025).

That paper provides a full explanation and example of the modern IL scenario‐planning process, to which the reader is referred. Importantly, in that paper, Derbyshire et al. (2025) show that participants in a scenario exercise, when asked to assign “extreme, yet still probable” values to the focal outcomes of a scenario's underlying causal factors, assign less extreme outcomes than when they are asked to assign values that are surprising. Although not a direct test of PST, aspects of which have been tested by others (Fisk and Pidgeon 1998), this provides supporting evidence that a focus on surprises extends and supports consideration of extremes, whereas probability constrains it.

## 6 Conclusion

Consider the most significant choices in one's own life. Whether it is to choose this or that subject to study at university, whether to marry, to have children or not, or many other similar decisions. Such decisions are not seriable and divisible experiments on which a sample can be built to inform decision‐making. Rather, such decisions are one‐off, unique, and irreversible; they can never be repeated. Of course, people can marry several times, but never in the same circumstances.

Once made, such decisions have profound consequences because, far from merely updating the space of possible sequels, they destroy and remake that space anew. They generate new possibilities while deleting others, thereby both augmenting and pruning the branching array of potential paths into the future, but in ways that will only become completely known over the fullness of time. By making such decisions, we turn the kaleidoscope, destroying the pattern its shapes had previously fallen into and generating another just as unique, yet also just as impermanent. At any given crucial decision juncture, the question is, therefore, what will be the pattern of our life as it emerges from the next turn of the kaleidoscope?

In the context of risk analysis, crucial decisions pose a profound challenge because, given all the knowledge that would be revealed as relevant to it over infinite time, a risk analysis must be based on the cross‐section available at the solitary present moment of its undertaking. Inevitably, possibilities that only later come into focus, or which are only subsequently generated, will be excluded from the analysis. To make a risk analysis complete, therefore, requires the use of a residual, or, put alternatively, a catch‐all category for scenarios that are currently unknown.

However, the use of a residual poses a profound challenge for probability‐based approaches, including those grounded in subjective probability, such as Bayesian learning and inference. The problem is that by its very nature (i.e., as it is composed of unknowns), it is impossible to know ex ante what the residual's probability should be. The potential is to underestimate its size because any probabilistic analysis emphasizes what is known rather than what is unknown. This underestimation would make it difficult to incorporate new possibilities into the analysis as they emerge from the residual over time. Shackle's PST can help in overcoming this problem. Its measure of uncertainty is nonadditive and so can accommodate new possibilities as they emerge over time without affecting the uncertainty assigned to those already known and considered.

By way of final conclusion, it is worth noting that, as much as PST offers a practical tool and a theoretical foundation for the uncertainty‐based perspective on risk, perhaps its still more important contribution is its overarching and underlying philosophy. PST reflects Shackle's ontology, which is the antithesis of the *E*[*X*]‐based thinking that has come to dominate across many fields today. If one were needed, PST provides the antidote to the now‐dominant *E*[*X*]‐based mindset and approach, which has its purposes but also has many regularly overlooked deficiencies.

Towards the end of his career, because of his emphasis on subjectivity and uncertainty, the accusation leveled at Shackle was one of nihilism. This pushed him and PST further to the margins, as *E*[*X*]‐based thinking continued its ascendancy to dominance. But consider what is truly nihilistic. Is it to recognize the impossibility of ever having absolute foreknowledge of the future and all it holds in store, and then to learn to live with and overcome this uncertainty? Or is it instead the denial of uncertainty by assuming the future to be completely knowable in all its important aspects?

In its turn towards an uncertainty‐based perspective, and perhaps even under its more traditional perspective, many risk analysts would agree that denying the full extent of uncertainty is nihilistic. That is why the very first paper in this journal recognized the impossibility of closing the future by exhaustively listing all its possibilities. Therefore, if the denial or downplaying of uncertainty is nihilistic, then the need is to use tools that both recognize the full extent of uncertainty and can cope with it. It is hoped the reader is now persuaded that G. L. S. Shackle's PST has much to offer in that regard.

## Conflicts of Interest

The author declares no conflicts of interest.

## Data Availability

Data sharing not applicable to this article as no datasets were generated or analyzed during the current study.

## Endnotes

1 Unknowledge is the term Shackle (1955a, 1961) used to describe the inverse of knowledge. It refers to what an analyst does *not* presently know about a focal event, outcome, or activity, and its potential consequences and triggers (Derbyshire and Aven 2025).

2 Shackle may have got his concept of the “kaleidic” society from Keynes (1930, 81), who specifically used the metaphor of a kaleidoscope to describe the influence of monetary changes on price levels (Madsen 2016).

3 The words “scenarios” and “possibilities” are used interchangeably throughout.

4 Nonadditive measures of uncertainty generalize probabilities by replacing the additivity condition with a less restrictive and more flexible one (Torra 2023). The additivity condition in probability theory is a fundamental principle stating that the probability of the union of mutually exclusive (i.e., disjoint) events is equal to the sum of their individual probabilities. Several nonadditive measures of uncertainty have been proposed by various scholars, and that associated with Shackle—that is, potential surprise—is just one (Basili and Zappia 2009b).

5 There is the danger in this paper of conflating the roles of risk analysts, decision‐makers, and regulators and, relatedly, of conflating risk analysis, decision analysis, and expected utility theory. To head off this danger, it should be noted that Paté‐Cornell and Dillon (2006) imply that probabilistic risk analysis and risk management (which may involve decisions by regulators and other stakeholders) are distinct. The risk analyst may not be involved in decisions made to manage risks. However, given the need for twofold inference and the recursivity this entails, and considering the nature of decisions representative of crucial experiments, as discussed herein, there may be an argument for risk analysts, decision‐makers, and policymakers responsible for risk management to work much more closely with each other.

6 The oil crises of the 1970s, precipitated by the decision of OPEC nations to reduce supply, popularized the use of the decision aid known as scenario planning (Derbyshire, Dhami, et al. 2023), which we later discuss as a method for operationalizing PST. Perhaps another oil crisis, this time precipitated by war, will popularize PST.

7 As described by Basili and Zappia (2009a, 250, Endnote 5), seriability and divisibility are the characteristics Shackle attributed to trials in a controlled environment, such as the rolling of a die, and to single events that can be pooled together to form a sample, such as the claims an insurance company must confront. Shackle contended that probability can only deal with experiments constituted by a set of repeatable trials of this kind. His focus was instead on experiments of a wholly different type because they are unique and unrepeatable.

8 This is why Lindley (1982) formulates what he calls “Cromwell's rule,” which implies that one should never assign a prior probability of exactly 0 or 1 to contingent propositions, except for logical impossibilities or certainties. The name comes from a 1650 letter from Oliver Cromwell to the Church of Scotland, pleading, “I beseech you, in the bowels of Christ, think it possible that you may be mistaken.”

9 This emphasis on imagination is evident in that Shackle (1949a, 2) stated his concern to be the “form of wishful thinking that is by no means stupid or even illogical; but is, indeed, the natural and reasonable response of human nature to intractable uncertainty.”

10 Philosophically minded readers might note the similarity between this dilemma and that of the so‐called “trolley problem” (Thomson 1976).

## Appendix 1

### 1.1

The following is taken from a chapter titled “Potential surprise axiomatized” (Shackle 1961, 79–85). It is stated almost verbatim as set out in that chapter, starting with the comments directly below.

By a hypothesis is meant any suggested answer to any question, and by the contradictory of this hypothesis is meant the hypothesis that the suggested answer will turn out to be wrong. By *rival hypotheses* is meant two or more mutually exclusive hypotheses concerning the same question. By an *exhaustive set of rival hypotheses* is meant any set of heads under which the individual feels certain that the true answer to some particular question, the true outcome of some particular experiment, when it shall become known, will prove to be classifiable. Any set of rival hypotheses can evidently be made exhaustive by the addition to it of a *residual hypothesis*, defined as covering every particular hypothesis, whether the individual has formulated it precisely or not at all, and having any character which would exclude it from classification under the hypotheses in the initial set. Of the seventeen propositions that follow, numbers 1–9 are the postulates or initial propositions:

1. An individual's degree of belief in a hypothesis can be thought of as consisting of a degree of potential surprise associated with the hypothesis, and another degree of potential surprise associated with its contradiction.
2. Degrees of potential surprise can be zero or greater than zero. No meaning is attached to a degree of potential surprise less than zero. Degrees of potential surprise are bounded by that degree *ȳ*, called the absolute maximum of potential surprise, which signifies the absolute rejection of the hypothesis to which it is assigned—that is, absolute disbelief in the truth of the suggested answer to a question or the possibility of the suggested outcome of an “experiment.”
3. *Equality* between the respective degrees of disbelief felt by an individual in two hypotheses will then require, for its expression in terms of potential surprise, *two* statements, namely, that some given degree of potential surprise is attached to both hypotheses, and that some given degree is attached to the contradictories of both.
4. The degree of potential surprise associated with any hypothesis will be the least degree amongst all those appropriate to different mutually exclusive sets of hypotheses (each set considered a whole) whose truth appears to the individual to imply the truth of this hypothesis.
5. All the members of an exhaustive set of rival hypotheses can carry zero potential surprise.
6. When *H* is any hypothesis, the degree of potential surprise attached to the contradiction of *H* is equal to the *smallest* degree attached to any rival of *H*.
7. Let $y_{A}^{B}$ be the degree of potential surprise assigned to a hypothesis *B* when $y^{A}$ is the degree assigned to a hypothesis *A*, and let $y_{0}^{B}$ be the degree assigned to *B* when $y^{A}$ = 0. Then $y_{A}^{B}$ is not greater than the greater of $y^{A}$, $y_{0}^{B}$.
8. Any hypothesis and its contradictory together constitute an exhaustive set of rival hypotheses.
9. At least one member of an exhaustive set of rival hypotheses must carry zero potential surprise. However, it is possible for all the rival hypotheses, which are, to any degree, particularized or specified, to carry potential surprise greater than zero because only the residual hypothesis carries zero potential surprise.

The remaining propositions 10–17 are theorems:

10 From (8) and (9), it follows that the degree of potential surprise that a person associates with the contradiction of a hypothesis cannot be greater than zero unless the degree he associates with the hypothesis is zero, and vice versa.
11 From (5) and (8), it follows that a hypothesis and its contradictory can each carry zero potential surprise (notwithstanding that the contradictory may itself be capable of being resolved into two or more mutually rival hypotheses).
12 Let *H* be any hypothesis. Then from (6), it follows that the degree of potential surprise attached to the contradictory of *H* cannot be greater than zero unless *every hypothesis rival to H* carries some degree of potential surprise greater than zero.
13 From (10) and (12), the contradictories of two rival hypotheses (i.e., two mutually exclusive hypotheses both concerning the same question) cannot both carry a degree of potential surprise greater than zero. For if *H_A_* and *H_B_* are two rival hypotheses, and the contradictory of *H_A_* carries some degree greater than zero of potential surprise, this implies by (12) that *H_B_* carries some degree greater than zero of potential surprise; but (10) implies that the contradictory of *H_B_* cannot then carry any degree of potential surprise greater than zero.
14 From (3) and (13), it follows that if *equal degrees of belief* are felt in two rival hypotheses, the contradictories of both must carry zero potential surprise. The highest degree of *equal belief*, which can be reposed in two rival hypotheses, consists in assigning to both of them, and to the contradictories of both of them, zero potential surprise.
15 Let $H_{1}^{1}$, $H_{2}^{1}$,…, $H_{n}^{1}$ be an exhaustive set of rival hypotheses concerning a first question, and let them carry respective degrees of potential surprise $y_{1}^{1}$, $y_{2}^{1}$,…, $y_{n}^{1}$. Let *Y* be the degree of potential surprise assigned to a hypothesis $H^{2}$ suggested in answer to a second question. In addition, let $y_{i}^{2}$ be the degree that will be assigned to $H^{2}$ if $H_{i}^{1}$ shall prove true. There are then two cases. First, if the $y_{i}^{2}$ are all equal, we assert that *Y *= $y_{i}^{2}$. For by (9) at least one of the $y_{i}^{1}$, say $y_{k}^{1}$, is zero, and by (7), *Y* will then not be greater than $y_{k}^{2}$. As the set $H_{i}^{1}$ is exhaustive, at least the degree $y_{i}^{2}$ must be assigned to $H^{2}$. Thus *Y* = $y_{i}^{2}$.
16 Second, if the $y_{i}^{2}$ are *not* all equal, we assert that *Y* will be equal to the *least* degree, which can be found amongst the complete set consisting of the *greater number of each pair* $y_{i}^{1}$, $y_{i}^{2}$. For by (7), *Y* cannot be greater than the greater member of some pair amongst this set. But by (4), it will be the least amongst these greater members.
17 Let symbols have the meanings given them in (16). Then the individual will attach a degree *greater than zero* of potential surprise to the hypothesis that, when the answer to the first question shall have become known, he will *reduce* the degree *y* of potential surprise he attaches to any hypothesis $H^{2}$ concerning the second question. For to assert the contrary would be to assert that there is some hypothesis $H_{K}^{1}$ to which the individual initially assigns potential surprise $y_{K}^{1}$ equal to *zero*, and that the degree of potential surprise which this answer to the first question implies for the particular answer $H^{2}$ to the second question is *less* than the degree *y* of potential surprise *initially* assigned, in *ignorance* of the answer to the first question, to $H^{2}$. But this would involve a contradiction. For out of the pair $y_{K}^{1},\;y_{K}^{2}$ we have $y_{K}^{1}=0$, and therefore by (2) $y_{K}^{2}$ is not less than $y_{K}^{1}$. But if $y_{K}^{2}$ can stand for the greater of the pair $y_{K}^{1},\;y_{K}^{2}$, then it appears in the set amongst which, by (16), the *least* member is *y*, namely, the degree of potential surprise initially assigned to $H^{2}$ $y_{K}^{2}$ cannot, therefore, be less than *y*.
